# How to Supplement Mentalist Evidentialism: What Are the Fundamental Epistemological Principles?

**DOI:** 10.1111/theo.12394

**Published:** 2022-02-09

**Authors:** Philipp Berghofer

**Affiliations:** ^1^ Department for Philosophy University of Graz Graz Austria

**Keywords:** explanationism, evidentialism, internalism, mentalism, phenomenology

## Abstract

Evidentialism and mentalism enjoy much popularity. In fact, mentalist evidentialism is often considered the most plausible internalist approach towards epistemic justification. However, mentalist evidentialism does not amount to a comprehensive theory of epistemic justification. In their attempt to complete their epistemological system and to answer the question of *why* experiences are justifiers, Conee and Feldman supplement mentalist evidentialism with *explanationism*. They take *principles of best explanation* to be the fundamental epistemic principles. In this paper, I show that explanationist mentalist evidentialism is plagued by severe shortcomings. What is more, I argue for an alternative in the spirit of Conee and Feldman's internalism that avoids the problems of explanationism, offering a straightforward commonsense account of epistemic justification. The fundamental epistemological principles are *phenomenological* principles.

## 1 INTRODUCTION

In contemporary debates, Conee and Feldman's mentalist evidentialism is considered one of the most promising internalist accounts of epistemic justification. Mentalist evidentialism holds that evidence determines justification and that one's justification supervenes on one's mental states. Concerning the question of *which* mental states are epistemically significant, Conee and Feldman single out *experiences* as the primary justifiers. One's experiences are one's ultimate evidence. This fits well with current movements that might be labelled experience‐first epistemologies (cf. Dougherty & Rysiew, 2014; cf. also Silins, 2014 and Smithies, 2014). Importantly, Conee and Feldman explicitly pose the question as to *why* experiences justify. What gives experiences their justificatory force? This is the question I am mainly concerned with. Conee and Feldman answer this question by subscribing to *explanationism*. Explanationism says that a person S is justified in believing proposition *p* at time t iff *p* is part of the best explanation available to S at t for the total evidence S has at t. Concerning the justificatory force of *experiences*, this means that experiences play a part in justifying propositions iff these propositions are part of the best explanation available to the subject for why the subject is having these experiences. Explanationism has been prominently championed by scholars such as Kevin McCain and Matt Lutz, but it faces many objections.

In this paper, I propose an alternative to supplementing mentalist evidentialism with explanationism. This alternative is based on a *phenomenological* conception of experiential justification (PCEJ). Contra Conee and Feldman, I argue that the fundamental epistemic principles are not principles of best explanation but phenomenological principles. According to PCEJ, justification‐conferring experiences gain their justificatory force by virtue of their distinctive phenomenology. The objective of this paper is to show that my phenomenological account enjoys a number of crucial advantages over an explanationist account. This is achieved in the sections 4 and 6. Whereas section 4 highlights shortcomings of the explanationist account, section 6 demonstrates the respective virtues of the phenomenological account. Section 4 and section 6 are structured analogously. For instance, whereas section 4.1 argues that the explanationist account is unsatisfying from an internalist perspective, section 6.1 clarifies that the phenomenological account is straightforwardly internalist.

It is to be noted that it is not its objections to explanationism that make this paper a novel and original contribution. Some of my objections to explanationism are only refinements of well‐known arguments. The novelty lies in demonstrating how mentalist evidentialism can be supplemented by PCEJ and how such a phenomenological supplementation leads to advantages over an explanationist supplementation. In particular, I show that internalists with sympathies for an experience‐first epistemology should prefer my phenomenological account. This is not only because it ascribes to experiences a truly foundational role such that experiences justify without any recourse to best explanations but also because it leads to a more adequate account of experiential justification. However, it is to be noted that my phenomenological supplementation is more limited in its scope and ambition. Whereas the explanationist supplementation deals with epistemic justification as such (including inferential justification), I restrict myself to experiential justification and the question of what it is that gives experiences their justificatory force.

## 2 MENTALIST EVIDENTIALISM

Evidentialism was introduced into current epistemology in a paper of the same name by Conee and Feldman in 1985. The basic idea of evidentialism is “that the epistemic justification of a belief is determined by the quality of the believer's evidence for the belief” (Conee & Feldman, 2004, 83). In order to provide a more precise account, Conee and Feldman have determined the relationship between justification and evidence in terms of a supervenience claim: “The epistemic justification of anyone's doxastic attitude toward any proposition at any time strongly supervenes on the evidence that the person has at the time” (Conee & Feldman, 2004, 101).

Conee and Feldman do not claim that their evidentialism is an exciting or novel thesis. Epistemologists sympathetic to evidentialism have stressed that British thinkers such as Locke, Hume, Clifford, and particularly Reid have championed or at least anticipated evidentialism (cf. McCain, 2014, 1; Rysiew, 2011; Dougherty, 2018). However, whereas evidentialism is an informative thesis about the systematic role of evidence that has been endorsed by many past and present philosophers, *it can only be the first step towards a full‐fledged epistemological system*. Most importantly, evidentialism as such does not tell us anything about the *nature of evidence*. What is evidence and what does it mean for a subject to possess evidence? This is why it is occasionally stated that evidentialism is not so much in danger of being refuted as in danger of being a trivial claim, “a mere truism” (Dougherty, 2018, 39 f.).

In most of their writings, Conee and Feldman say surprisingly little about the nature of evidence. However, in Conee & Feldman, 2008, they are very well aware of the limits of evidentialism. What is more, this is also where they say most about the nature of evidence and about the details of their preferred epistemological system. The first thing to note is that they combine their evidentialism with a position they term *mentalism*: “We are mentalist evidentialists. That is, we think that one's justifying evidence supervenes on the totality of one's mental states” (Conee & Feldman, 2008, 99). Mentalism is Conee and Feldman's preferred version of epistemic internalism. Epistemic internalism is most often associated with a form of *access internalism*. However, Conee and Feldman have argued for capturing the basic idea of internalism differently. According to them, “internalism is the view that a person's beliefs are justified by the things that are internal to the person's mental life” (Conee & Feldman, 2004, 55). More precisely, they introduce their mentalism as follows:

Mentalism: “The justificatory status of a person's doxastic attitudes strongly supervenes on the person's occurrent and dispositional mental states, events, and conditions.” (Conee & Feldman, 2004, 56)

They point out that their mentalism implies the following thesis:

Mentalism*: “If any two possible individuals are exactly alike mentally, then they are alike justificationally, e.g., the same beliefs are justified for them to the same extent.” (Conee & Feldman, 2004, 56)

Alternatively, mentalism has been characterised as

Mentalism’: “If e is evidence for S that *p*, e is a mental state of S's.” (Appley & Stoutenburg, 2016)

Externalists typically deny access internalism as well as mentalism in its various versions.^1^ Mentalism is a controversial but popular view in current epistemology. One obvious advantage of mentalism is that, in contrast to access internalism, it is not in danger of leading to a vicious regress of justification. Concerning the different versions of mentalism, it is to be noted that Mentalism’ is a stronger (and more informative) claim than Mentalism. Mentalism is a supervenience claim that does not specify the nature of our evidence or justifiers. Mentalism’, by contrast, explicitly identifies our mental states as our evidence. Because in this paper I am mainly concerned with the nature of our justifiers and the question of what gives our justifiers their justificatory force, I often use “*mentalism*” in the following sense: If some J is a justifier, J has to be a mental state. To be sure, mentalism is only committed to the view that every justifier is a mental state but not to the claim that every mental state is a justifier. Some unconscious, indeterminate state of anxiety may be a mental state, while not being a justifier. One important task mentalists face is to specify *what mental states exactly* are supposed to constitute our (primary) source of justification.

In Conee & Feldman, 2008, they clarify that the mental states that constitute our evidence are *experiences*. “Experience is our point of interaction with the world – conscious awareness is how we gain whatever evidence we have” (Conee & Feldman, 2008, 87). They specify the fundamental role of experiences as follows: “We believe that all ultimate evidence is experiential evidence” (Conee & Feldman, 2008, 86). This means that, so far, we can define Conee and Feldman's version of mentalist evidentialism as follows:

CFME1 (evidentialism): Evidence determines justification.

CFME2 (mentalism): One's justification supervenes on one's mental states.

CFME3 (experience‐first epistemology): One's experiences are one's ultimate evidence.

Every mentalist evidentialist must subscribe to CFME1 and CFME2. CFME3 singles out experiences as those mental states that play the fundamental epistemic role. Theoretically, mentalist evidentialists could deny CFME3 and ascribe this fundamental epistemic role to a different type of mental states (e.g., to beliefs). However, I take it that CFME3 is the best answer to the question: Which mental states are one's ultimate justifiers? There is one crucial question left that we will discuss in the next section, namely: *Why do experiences justify*; what gives them their justificatory force? The answer to this question is where I differ from Conee and Feldman and the main objective of this paper is to demonstrate the advantages of my answer over the answer of Conee and Feldman (CFME4 as specified in the next section). Before we turn to the differences between me and Conee and Feldman, let me briefly emphasise why we should subscribe to CFME1‐CFME3.

Conee and Feldman's version of mentalist evidentialism enjoys much popularity. Evidentialism is a plausible claim with a long history that many consider trivially true. Mentalism is motivated by internalist thought experiments such as the new evil demon problem (cf. section 6.1). Conee and Feldman's distinctive claim that experiences are one's ultimate evidence fits well with current movements in epistemology that might be labelled *experience‐first epistemologies* (cf. Dougherty & Rysiew, 2014; cf. also Silins, 2014; Smithies, 2014). Of course, mentalist evidentialism is particularly attractive to epistemologists with internalist and foundationalist sympathies. However, one might argue that, vice versa, the main reason to endorse internalism and foundationalism is how well they fit with the plausible claim that experiences are our ultimate justifiers (cf. Smithies, 2014). To put it differently, CFME1‐CFME3 complement each other perfectly.

The most satisfying feature of CFME1‐CFME3 is how well they handle everyday cases of justification. When I see a black laptop in front of me, my laptop experience is my evidence, that is, what justifies me in believing that there is a black laptop, and any internal twin of mine is equally and for the same reason justified in believing so. It is a virtue of mentalist evidentialism to stand in the tradition of Reidian Commonsensism and to provide a straightforward account of epistemic justification. However, CFME1‐CFME3 do not constitute a comprehensive epistemological system. For instance, they do not provide an answer to the question of *why* experiences have justificatory force. The objective of this paper is to supplement mentalist evidentialism by providing a straightforward answer to this question that fits well with the overall internalist and foundationalist spirit expressed by Conee and Feldman when they say: “Experience is our point of interaction with the world – conscious awareness is how we gain whatever evidence we have” (Conee & Feldman, 2008, 86). Interestingly, my account is very different from the one offered by Conee and Feldman themselves.

## 3 BEST EXPLANATIONISM

Experiences enjoy a special epistemic status. When I entertain the thought that

*p*: in the room next to me there is a table,

this act of thinking does not justify me in believing *p*. When I believe that *p*, the act of believing does not justify me in believing that *p*. The same is true for hoping that *p*, worrying that *p*, or imagining that *p*. However, when I enter the room and have a perceptual experience as of a table, plausibly, this experience provides immediate prima facie justification for believing that there is a table. But *why* do experiences justify; what gives them their justificatory force? This is a fundamental epistemological question that does not always get the attention it deserves. Conee and Feldman, however, explicitly pose this question: “In our view, if perceptual and memorial experiences are justifying (with the proper background in place), then, there is something about them that makes this the case. […] There must be a more illuminating truth about why the experiences are justifying” (Conee & Feldman, 2008, 97).

To put it differently, if experiences are justifying, there must an underlying epistemic principle that explains why this is so. Interestingly, the principle identified by Conee and Feldman seems to be somewhat at odds with the foundationalism^2^ and internalism indicated by CFME1‐CFME3.

We believe that the fundamental epistemic principles are principles of best explanation. Perceptual experiences can contribute toward the justification of propositions about the world when the propositions are part of the best explanation of those experiences that is available to the person. (Conee & Feldman, 2008, 97)

They clarify this statement by providing the following picture: “Thus, the general idea is that a person has a set of experiences, including perceptual experiences, memorial experiences, and so on. What is justified for the person includes propositions that are part of the best explanation of those experiences available to the person” (Conee & Feldman, 2008, 98). Accordingly, Conee and Feldman's version of mentalist evidentialism is an explanationist mentalist evidentialism. Their epistemological system is completed by adding CFME4.

CFME1 (evidentialism): Evidence determines justification.

CFME2 (mentalism): One's justification supervenes on one's mental states.

CFME3 (experience‐first epistemology): One's experiences are one's ultimate evidence.

CFME4 (explanationism): A person S is justified in believing proposition *p* at t iff *p* is part of the best explanation available to S at t for the total evidence S has at t.^3^

Explanationism faces many objections and does not enjoy much popularity. Its chief proponent in current debates is McCain, who defends the following advanced version of explanationism.

Explanationism*:

A person, S, with evidence *e* at *t* is justified in believing *p* at *t* iff at *t* S has

considered *p* and:

either (i) *p* is part of the best explanation available to S at *t* for why S has *e*,

or

(ii) *p* is available to S as an explanatory consequence of the best explanation

available to S at *t* for why S has *e*. (McCain, 2018a, 3042)

Clause (ii) is implemented in order to account for certain cases of inferential justification (cf., e.g., McCain, 2014, 62–65).

In what follows, I refer to an explanationist supplementation of mentalist evidentialism that subscribes to the theses CFME1‐CFME4 as explanationist mentalist evidentialism (EME). This is the position championed by Conee and Feldman and McCain. In the following section, I discuss some of the shortcomings of EME. These problems apply independently of whether the explanationist moment of EME is fleshed out by CFME4 or by explanationism*. In section 5, I will introduce my preferred supplementation of mentalist evidentialism, namely what I call a *phenomenological mentalist evidentialism* (as opposed to explanationist mentalist evidentialism). In section 6, I shall demonstrate the virtues and advantages of my phenomenological conception.

## 4 PROBLEMS FOR EXPLANATIONISM

In sections 4.1 and 4.2, I show that EME is neither truly internalist nor foundationalist. Of course, this is not necessarily a problem for EME (because what matters is whether our epistemological theories are correct not whether they have a certain label). But here we will already see that EME is not capable of providing the straightforward approach towards epistemic justification that one might expect. The sections 4.3, 4.5 discuss objections that are explicitly directed against the explanationist moment of EME. In section 6, I shall show how my phenomenological mentalist evidentialism can avoid these problems.

I want to emphasise that it is not the main objective of this paper to refute explanationism. I do not claim that the arguments in this section show that EME is indefensible. The main objective of this paper is to show that a *phenomenological* supplementation of mentalist evidentialism has many crucial advantages over an explanationist supplementation. The objective of this section is to shed light on the problems of explanationism and set the stage for section 6 in which I highlight the advantages of the phenomenological account. The present section and section 6 are parallel in structure in order to best highlight the virtues of the phenomenological account. Of course, this is not to say that phenomenological mentalist evidentialism cannot be attacked on different fronts. This paper does not aim at delivering a defence of the phenomenological account.^4^ The aim is to show that philosophers sympathetic to mentalist evidentialism should aim at a phenomenological supplementation instead of an explanationist one.

### 4.1 Is EME internalist?

There is a general consensus that mentalism is an internalist position. In fact, mentalism as well as accessibilism are considered the two main versions of internalism. But to consider mentalism as plainly internalist is a mistake. To see this more clearly, we first turn to evidentialism. Conee and Feldman have occasionally referred to evidentialism as a version of internalism (cf. Conee & Feldman, 2004, 53, 64, 73) and to view evidentialism as a version of internalism is also not uncommon in current debates (cf. Steup, 2016, section 2.3). But this is a mistake. Evidentialism as such does not tell us anything about the nature of evidence nor about what it means for a subject to possess evidence; thus, it is not specific enough to count as a version of internalism. Evidentialism is neither committed to internalism nor to externalism but is *compatible with both*. To see why, consider the following view: (1) evidence determines justification and (2) one's evidence consists exclusively of true propositions one believes such that these beliefs are produced by reliable processes. (1) makes this position evidentialist, and (2) makes this position clearly externalist. For a recent objection to the received view that evidentialism is committed to internalism, cf. Bergmann, 2018.

Concerning mentalism, we have seen that the guiding idea is “that a person's beliefs are justified by the things that are internal to the person's mental life” (Conee & Feldman, 2004, 55). Conee and Feldman flesh this out by saying that if two subjects “are exactly alike mentally, then they are alike justificationally” (Conee & Feldman, 2004, 56). However, it has been pointed out that thus defined mentalism is compatible with positions traditionally viewed as externalist (cf., e.g., Pritchard, 2011). This has already been noted by Conee and Feldman (cf. Conee & Feldman, 2004, chapter 3 and the afterword of chapter 3). Here, the prime example is Williamson's famous formula E = K. Williamson contends “that one's total evidence is one's total knowledge” (Williamson, 2005, 468). Because Williamson also argues that knowledge is a mental state, his knowledge‐first epistemology qualifies as a version of mentalism: One's evidence (i.e., justification) supervenes on one's knowledge, and because knowledge is a mental state, this means that justification supervenes on the mental. Similarly, consider disjunctivists who hold that (i) veridical perception is essentially distinct from illusion and hallucination even if these mental states are phenomenologically indistinguishable and (ii) only veridical perception is a source of justification but not illusion or hallucination. Even this disjunctivist position qualifies as mentalism.

To make sure that Williamson's view or disjunctivist accounts do not qualify as a version of mentalism, mentalism is often defined as follows: One's justification supervenes on one's *non‐factive* mental states. Because knowledge and veridical perception are factive, the embarrassment of classifying seemingly externalist positions as a version of internalism is thus avoided. However, to redefine mentalism as the view according to which justification supervenes on non‐factive mental states is ad hoc, particularly because the distinction between factive and non‐factive mental states is an *externalist distinction* that is not grounded in what is “internal to the person's mental life.”^5^

What is more, even if we define mentalism as implying that only non‐factive mental states are justifiers, it is still compatible with views that seem clearly externalist. For instance, one might claim that one's evidence solely consists of one's non‐factive but reliably produced experiences, and that they gain their justificatory force precisely by virtue of their reliability. This view is mentalist because it restricts one's evidence to one's (non‐factive) mental states, but it is also externalist because it amounts to a version of reliabilism.

Finally, one might argue that *explanationist* mentalist evidentialism is not truly internalist because, according to this position, not all justificatory work is done by the experiences. This is because if S is justified in believing that *p* based on a body of evidence consisting of S's experiences E_1_‐E_n_, then *p* and S's experiences must be related such that *p* is part of the best explanation available to S for why she is having E_1_‐E_n_. If this is supposed to mean that it must be objectively true that *p* is part of the best explanation and that it does not matter whether S in any sense recognises this, then this position is hardly internalist. On the other hand, if this is supposed to mean that S has some kind of disposition to have the seeming that *p* is (part of) the best explanation of the evidence she has (as required by McCain), then this position may be *access*‐internalist, but this position seems to lead to a vicious regress (cf. the end of section 4.5). Below, I will argue for a different kind of internalism, namely the claim that a factor *internal* to the experiences E_1_‐E_n_ gives these experiences their justificatory force. I take it that such a position fits even better the idea of Conee and Feldman that “internalism is the view that a person's beliefs are justified by the things that are internal to the person's mental life” (Conee & Feldman, 2004, 55). Importantly, my internalist position does not invite the regress problem (cf. the end of section 6.1).

### 4.2 EME is neither foundationalist nor coherentist

When Conee and Feldman emphasise that “[e]xperience is our point of interaction with the world” and insist that “all ultimate evidence is experiential evidence,” this suggests a foundationalist picture according to which experiences are the source of immediate justification and all inferential justification can somehow be traced back to experiential justification. This is reaffirmed when they explicitly address perceptual evidence (Conee & Feldman, 2008, section 2.1). Here they state that the visual experiences “E1–E*n* are at the beginning of the line” such that “they can be evidence for a proposition without one's having evidence for them” and that “they can justify without needing justification” (Conee & Feldman, 2008, 92). This seems to speak in favour of a foundationalist conception of experiential justification according to which experiences are a source of immediate prima facie justification. However, due to its explanationist element, EME tells a different story. As McCain elaborates, experiences cannot be an autonomous source of justification because what is needed in addition is the subject's background evidence that sustains the subject's disposition to have the seeming that the proposition in question is part of the best explanation of the subject's evidence (McCain, 2014, 120). McCain concedes that this is at odds with classical as well as with moderate forms of foundationalism. However, McCain emphasises that this is also at odds with traditional forms of coherentism because experiences play an important role and one's evidence is not restricted to one's beliefs. As he rightly notes, such an approach amounts to a hybrid between foundationalism and coherentism that has been called *foundherentism* (Haack, 1993) or weak foundationalism (BonJour, 1985). Similarly, Conee and Feldman say:

So it may be helpful to think of our view as a non‐traditional version of coherentism. The coherence that justifies holds among propositions that assert the existence of the non‐doxastic states that constitute one's ultimate evidence and propositions that offer an optimal available explanation of the existence of that evidence. (Conee & Feldman, 2008, 98)

Let us clarify the difference between this explanationist approach and a foundationalist approach by discussing a concrete example delivered by McCain. Assume a person called Sasha is looking at a red block such that her experience visually presents to her a red block. According to conceptions of experiential justification that I prefer and that have become increasingly popular, this experience immediately justifies Sasha in believing the proposition <there is a red block>.^6^ However, in the explanationist picture the situation is considerably more complicated:

Sasha's visual experience does not justify […] <there is a red block> on its own. Rather, her experience justifies [this proposition] only when Sasha possesses the relevant background evidence. She has to have background evidence that sustains her disposition for <there is a red block> to seem to her to be part of the best explanation of her evidence. This background evidence will include information about how the look of blocks, the look of red objects, and so on. (McCain, 2014, 120)

As pointed out above, I do not criticise EME for not qualifying as a form of foundationalism. Instead, there are at least three problems that emerge in this context. First, explanationism does not lead to a straightforward common‐sense approach towards experiential justification as opposed to some of its rivals (including the one I will argue for in section 6). Second, because EME amounts to a form of foundherentism or weak foundationalism, all the well‐known problems with this position also apply to EME.^7^ Third, the explanationist requirement that the subject has (the disposition for) a seeming that the proposition in question is part of the best explanation of her evidence is difficult to pin down precisely and leads to certain problems discussed below.

### 4.3 The charge of overintellectualization

As indicated above, the explanationist requirement that a person S must be disposed to have the “seeming that *p* is part of the best explanation available to S at *t* for why S has the evidence she does” (McCain, 2014, 78) is in danger of overintellectualizing of what it means to be experientially justified. It is plausible to assume that even certain higher animals as well as children possess some kind of experiential evidence, although they are not capable of reflective reasoning. To demand higher‐level requirements on justification is thus problematic. Versions of access internalism are typically accused of demanding implausible higher‐level requirements, and I take it to be a virtue of externalist approaches that they can avoid this problem. My aim is to establish an *internalist* version of epistemic justification that avoids such higher‐level requirements (cf. section 6.3). In the context of the accusation of overintellectualization, McCain points out that

[T]his sort of disposition [as demanded by the explanationist account] does not require one to have well‐developed concepts of ‘evidence’, ‘explanation’, ‘logical consequence’, or ‘entailment’. All that is required is the ability to understand something as an answer to a why‐question or the disposition to have a seeming that, for example, the truth of *p* and if *p*, then *q* ensure the truth of *q*. (McCain, 2014, 78)

I do not doubt that if a subject has some kind of inferential justification that requires logical reasoning, the subject must be capable of some kind of higher‐order reasoning. However, in the case of simple experiential justification, any higher‐order requirement seems problematic. For instance, I take it to be plausible that when a dog hears its favourite person approaching and then sees the person, the dog knows (or has justification to believe) that the person is there. However, because, plausibly, dogs lack the cognitive capabilities “to understand something as an answer to a why‐question” explanationist mentalist evidentialism has negative consequences I would like to avoid.

### 4.4 Degrees of justification made difficult

Justification comes in degrees. This is particularly obvious in the case of experiential justification. Seeing a physical object up close under good light conditions such that the object is presented to you clearly and distinctly provides more justification than seeing the same object from afar under bad light conditions such that the object is presented to you less clearly and less distinctly. Conee and Feldman believe that their explanationism can straightforwardly account for degrees of justification.

One's justification for a proposition can be of various strengths. Correspondingly, one's evidence varies in the strength of its support of different propositions. According to our explanatory coherence view of evidential support, this variation in strength of support derives from differences in how well the supported propositions explanatorily cohere with one's evidence. (Conee & Feldman, 2008, 98)

However, things are not that simple. The first question to be raised is whether explanatory coherence is to be understood objectively or subjectively. By an objective understanding, I mean that it is objectively true that the proposition *p* explanatorily coheres with the subject's evidence to the degree X (given that this explanation is available to the subject), and therefore the subject is justified to the degree X' in believing that *p*.

There are several problems with such an objectivist conception. First, and this is more of a sidenote, this objectivist account does not fit well with an internalist account; this factor of objective explanatory coherence is nothing that could be internal to the subject. Second, what would it mean to say that it is *objectively* true that the degree of explanatory coherence is of value X? Who or what sets the criterion? Finally, according to the objectivist conception, the epistemically normative notion of epistemic justification is grounded in another epistemically normative notion, namely best explanation. It is a virtue of externalist approaches to be able to pin down basic epistemic notions such as epistemic justification in non‐normative, objective terms such as truth or reliability. In this paper, I aim at an internalist conception that is able to account for experiential justification in entirely descriptive, non‐normative terms. Explanationism is not the way to go. (The objection that explantionism cannot account for epistemic justification in non‐normative terms is discussed in more detail in the following subsection.)

By a subjective understanding of explanatory coherence, I mean that it seems to the subject (or the subject is disposed to have the seeming) that the proposition *p* explanatorily coheres with the subject's evidence to the degree X, and thus the subject is justified to the degree X’ in believing that *p*. Such a subjectivist conception of epistemic justification may be particularly prone to the charge of overintellectualization raised in the previous section because it is unlikely that we have such specific seemings. However, I want to focus on another objection also based on experiential justification.

Imagine two subjects S1 and S2. S1 and S2 are mentally identical with two exceptions: (1) Whereas S1 is now having a clear and distinct perceptual experience as of a red table in front of her, S2 is now having a less clear and less distinct perceptual experience as of a red table in front of her. (2) For whatever reason, S1 has a comparably weak seeming that the proposition “there is a red table in front of me” explanatorily coheres with her evidence, whereas S2, for whatever reason, has a comparably strong seeming that the proposition “there is a red table in front of me” explanatorily coheres with her evidence. We stipulate that the seeming of S2 is stronger than the seeming of S1. The subjectivist understanding implies that S2 is better justified in believing that there is a red table in front of her. This is a highly counter‐intuitive consequence. I aim at an internalist approach that is not subjectivist in this sense. To be more precise, I aim at an approach according to which experiential justification is exclusively determined by factors internal to the experiences, not including how the subject feels about what is presented to her nor including what seemings the subject has concerning relations of explanatory coherence. (Such seemings that concern relations of explanatory coherence, however, may contribute to the overall justification a subject has, but not to the immediate justificatory force of experiences.)

### 4.5 Is epistemic justification grounded in non‐normative terms?

As discussed at the beginning of section 3, Conee and Feldman pose the question of why experiences are justifiers. They want to unveil the underlying epistemic principle that grounds experiential justification. To say that an experience has justificatory force or that a belief is epistemically justified is to make a normative claim. When aiming at explaining on a more fundamental level why experiences have justificatory force, it is required that this explanation is fleshed out in terms that are clearer or better understood than the terms it seeks to explain. Concerning epistemic justification, it is often claimed that such an explanation must not include epistemic or normative terms (cf. Steup, 2018 and the references in Steup, 2018, 362). In this context, Matthias Steup says: “Hence, for a satisfying and truly informative theory of epistemic justification, the goal is to ground epistemic evaluation in conditions that are completely factual, that is, non‐normative” (Steup, 2018, 362; cf. also Beddor, 2015, 1860).

Accordingly, it is a virtue of externalist conceptions of epistemic justification when they account for epistemic notions in non‐normative terms. For instance, a reliabilist conception specifically addressing experiential justification may look like this:

Reliabilism: Your experience E representing that *p* immediately justifies you in believing that *p* iff E is the product of a reliable process.

Is there an internalist conception of experiential justification that also enjoys the virtue of pinning down the epistemic notions of experiential justification in non‐normative, objective terms? Does explanationist mentalist evidentialism offer such a non‐normative approach? Steup (2018) provides detailed arguments that it does not. Here I will not discuss Steup's arguments. However, I want to emphasise two points. First, because explanationism accounts for epistemic justification in terms of “the best explanation” that is available to the subject for why she has the evidence she has, it is obvious that there is at least a prima facie problem for explanationist accounts. Second, when Steup raises the worry of how the explanationist availability condition could be fleshed out in non‐epistemic terms, McCain responds that his explanationism grounds “availability in dispositions to have seemings” (McCain, 2018b, 388). The idea is that because “seemings are non‐normative mental states, dispositions to have such non‐normative mental states also appear to be non‐normative” (McCain, 2018b, 388).

This brings us back to the dilemma discussed in the previous section. Explanatory coherence can be understood in an objective or in a subjective sense. In the objective sense, this means that *p* objectively is part of the best explanation available to the subject. But this means to flesh out one epistemic notion (justification) via another epistemic notion (best explanation). If so, then it seems that EME cannot be considered a fundamental epistemic theory.

In the subjective sense, it may not be true that *p* really is part of the best explanation available to the subject for why the subject has the evidence she has; it suffices that it seems to the subject that it is the best explanation. This subjectivist approach avoids the problem raised by Steup (because then all that matters is the phenomenal character of the seeming the subject is disposed to have and this character can be fleshed out descriptively in non‐normative terms); however, it leads to the problem discussed in the previous section. It seems that while McCain champions the subjectivist approach, Conee and Feldman champion the objectivist approach.

One final note concerning the explanationist availability condition. Even proponents of explanationism concede that “[a] precise account of this availability is difficult to develop” (Conee & Feldman, 2008, 98). Particularly if the requirement that the subject must have the disposition to have the seeming that the proposition in question is part of the best explanation available to the subject for why the subject has the evidence she has is combined with evidentialism and mentalism, this seems to imply a vicious regress. This is because the disposition to have such a seeming is itself a mental state that is justificationally relevant. Thus, according to mentalist evidentialism, this disposition is itself a piece of evidence. Appley and Stoutenburg have argued that this disposition is an emerging *new* piece of evidence such that a new dispositional seeming is required, and so on ad infinitum (Appley & Stoutenburg, 2016).^8^ In my experience, researchers sympathetic to EME are not much impressed by the regress problem. This is because it is often assumed that *any* internalist positions faces this problem.^9^ But this is not true. Below I will introduce a supplementation of mentalist evidentialism that is clearly internalist but is not in danger of leading to a vicious regress (cf. particularly section 6.1).

I do not argue that the objections against an explanationist supplementation of mentalist evidentialism that we encountered in this section are conclusive evidence that EME is untenable. However, we have seen that EME is plagued by severe shortcomings and that philosophers with internalist and foundationalist sympathies in particular should look for an alternative supplementation of mentalist evidentialism. It is the objective of the following two sections to introduce and motivate such an alternative.

## 5 PHENOMENOLOGICAL MENTALIST EVIDENTIALISM

I aim at a supplementation of mentalist evidentialism that is internalist, foundationalist, and provides a straightforward commonsense approach towards experiential justification. In the spirit of Conee and Feldman, I pose the question of why certain experiences are justifiers. In contrast to Conee and Feldman, I shall provide a straightforward answer to this question. My approach is in the spirit of, for example, Chudnoff, 2013, Church, 2013, and Pryor, 2004. The idea is that certain experiences gain their justificatory force from their distinctive phenomenology.^10^ I call this the phenomenological conception of experiential justification (PCEJ). More precisely, the claim is

PCEJ: Certain experiences have a distinctive, justification‐conferring phenomenology, and if an experience E has such a justification‐conferring phenomenology with respect to proposition *p*, E, by virtue of its phenomenology, provides immediate prima facie justification for believing that *p*.

PCEJ implies that certain experiences can confer justification and provides an answer to the question of *why* certain experiences can confer justification. PCEJ closely connects epistemology and philosophy of mind. This conception is *phenomenological* in a twofold sense. On the one hand, it puts a focus on the way things appear to us within experience and by doing so links an experience's justificatory force to its phenomenology; on the other hand, such an approach can be found in the phenomenology of Edmund Husserl.^11^

Different versions of PCEJ differ in how they characterise the justification‐conferring phenomenology of justifying experiences. The most detailed version of PCEJ can be found in the works of Elijah Chudnoff (cf., e.g., Chudnoff, 2013). My own preferred version of PCEJ differs in important respects from Chudnoff's account (cf. Berghofer, 2020b). However, the objective of this paper is not to provide a detailed account of the justification‐conferring phenomenology of certain experiences. Instead, for the present purpose, it suffices to provide a general outline of what I take the distinctive phenomenology of *perceptual* experiences to consist in. The idea is that perceptual experiences have a *presentive* character. Perceptual experiences do not simply represent their contents/objects; they present them in a distinctive manner. When I look at the laptop in front of me, my perceptual experience presents the laptop as bodily present. I am visually aware of the laptop, of its form, its colour, its keyboard, etc. Importantly, perceptual experiences only justify contents with respect to which they have this presentive character. My laptop experience, for instance, does not justify me in believing that the laptop was acquired on a specific date. My visual experience, for whatever reason, may make it seem to me or push me towards believing that the laptop was purchased on November 11, 2011. However, because it does not have a presentive phenomenology with respect to this content, it does not justify believing this content.^12^

To gain some initial motivation for PCEJ, just think about your everyday perceptual experiences. Most philosophers, whether or not they are sympathetic to PCEJ, agree that when you have the perceptual experience that there is a tree in your yard, then you are (prima facie) justified in believing that there is a tree in your yard. It also seems uncontroversial that perceptual experiences, like this tree experience, have a distinctive phenomenology that distinguishes them from other mental states like thinking, believing, imagining, or wishing. You might *think about* a tree in your yard; you might *believe* or *imagine* or *wish that* there is a tree in your yard. You can do all these things with your eyes closed. But when your eyes are open and you *see* a tree in your yard, this experience is *phenomenologically clearly different* from the aforementioned mental states. In this example, the act of seeing is epistemologically as well as phenomenologically distinguished from the other mentioned mental states. Epistemologically, because it is the only one that has justificatory force concerning its content. Phenomenologically, because it is the only one that has a “presentive” phenomenology concerning its content. PCEJ implies that this phenomenological–epistemological parallelism is no coincidence. Below we will see in more detail that PCEJ amounts to an initially plausible commonsense conception of experiential justification that straightforwardly accounts for everyday cases (why do clear and distinct experiences have more justificatory force than vague and obscure ones?) as well as popular internalist thought experiments (e.g., the new evil demon problem).

One way of clarifying what it means that an experience has a presentive phenomenology, at least with respect to perceptual experiences, is by pointing to what I call the “seeming awareness of the experience's object.” An experience's object is the object your experience is intentionally directed at. An experience E has the phenomenal character of seeming awareness with respect to an object O (or proposition *p*) if E makes it seem to you that you are aware of O. If your visual experience has a presentive phenomenology concerning the proposition that there is a table in front of you, it seems to you that you are visually aware of a table in front of you. I say *seem* to be aware in order to indicate two things. Firstly, not only veridical experiences but also illusions and hallucinations can have this phenomenal character. If you have a perfect hallucination of a table in front of you, it seems to you that you are aware of a table in front of you. Secondly, if it seems to you that you are aware of O, you are intentionally directed towards O as O. This means if it seems to you to be aware of Clark Kent, you are intentionally directed towards Clark Kent as Clark Kent, not as Superman. Literally speaking, you might be aware of Superman, but your experience has the phenomenal character of seeming awareness only with respect to Clark Kent, not Superman. In short, by having a perceptual experience it does not merely seem to one that *p*, but it seems to one that one is perceptually aware of *p*.

What matters epistemologically, according to my account, is how an experience presents its content, not how an experience makes you feel about its content. An experience's justificatory force is *grounded* in its phenomenology in the sense that what is given to you within experience and how it is given to you within experience determines the experience's justificatory force. If an experience has a justification‐conferring phenomenology, it justifies believing precisely those propositions with respect to which it has its justification‐conferring phenomenology. Perceptual experiences have a justification‐conferring phenomenology. I used the seeming terminology to account for the phenomenology of perceptual experiences due to the popularity of Michael Huemer's principle of phenomenal conservatism (PC) (Huemer, 2001, 2007). This allowed me to best contrast my account with its current rivals.

Accordingly, my PCEJ bears many systematic resemblances to Huemer's PC, according to which every seeming is a source of immediate prima facie justification. However, there are also a number of subtle but important distinctions. Let me briefly summarise these distinctions. First, Huemer's position is not evidentialist. Huemer opposes evidentialism; accordingly, his position is not spelled out in terms of evidence. Second, PC does not state that seemings are justifiers *by virtue* of their distinctive phenomenology; thus, PC does not qualify as a version of PCEJ. Of course, one could easily modify PC such that it amounts to a version of PCEJ (by simply stating that seemings gain their justificatory force by virtue of their distinctive seeming phenomenology). Importantly, there are still significant differences in how the justification‐conferring phenomenology is spelled out. This is because, third, I do not believe that the phenomenology of perceptual experiences is best spelled out as making it seem that something is the case. This is a descriptive point. I do not believe that the phenomenology of perceptual experiences is adequately described in terms of making it seem that *p*. Fourth, I do not believe that *any* experience has justificatory force simply by making it seem that *p*. This is an epistemological point. This point is motivated by the many counterexamples that have been raised against PC. These counterexamples, typically, exploit that it is counterintuitive to say that one is immediately justified in believing that *p*, simply because it seems to one that *p*, even if *p* is not presented experientially. This is why I would prefer a version of PCEJ according to which what matters epistemologically is how an experience presents its content, not how an experience makes you feel about its content.

Fifth, similar to Huemer, I believe that there are various types of justification‐conferring experiences such as perceptual experiences, rational intuitions, and introspective experiences. Importantly, I believe that each type of justification‐conferring experience corresponds to a distinctive type of justification‐conferring phenomenology. Rational intuitions can have a presentive character with respect to necessary truths, introspective experiences with respect to one's own mental states, and perceptual experiences with respect to physical objects. But they each present their respective contents/objects in importantly different manners. Accordingly, I believe that it would be phenomenologically as well as epistemologically inadequate to say that, for example, perceptual experiences and rational intuitions justify by virtue of the same type of phenomenology. This differentiation is in stark contrast to Huemer's PC that holds that perceptual experiences and rational intuitions both justify qua being seemings. Neither do I believe that the seeming characterisation adequately captures the distinctive phenomenology of perceptual experiences, nor do I believe that the seeming characterisation adequately captures the distinctive phenomenology of any other type of justification‐conferring experience, nor do I believe that the various types of justifications‐conferring experiences can be subsumed under the same phenomenological characterisation. The reason I focus on perceptual experiences is that this is the type of experience most often discussed in the literature I am engaging with. In particular, it is the prime example discussed by Conee and Feldman. It would go beyond the scope of the paper to discuss the phenomenology of other types of justification‐conferring experiences in sufficient detail.

One more clarification is in order: In the context of an experience's justification‐conferring phenomenology, it has become popular to talk about an experience's “phenomenal force” (Pryor, 2000, 547, note 37). Huemer refers to the distinctive phenomenal character of seemings as “forcefulness” and Tolhurst characterises it as “the feel of truth.” In this context, Teng states that perceptual experiences “assure the subjects of the contents' truth” (Teng, 2018, 641) and that “dogmatists seem to take phenomenal force as analogous to the assertiveness of testimony” (Teng, 2018, 641). Such characterizations are misleading at best, and I would like to point out that what I understand by presentive phenomenology is *clearly distinct* from the “assertiveness of testimony.” It is *no mere feeling* of truth, and it is not forceful in the sense of forcing one *psychologically* to believe its content. It is only forceful in the sense that the presence of the experience's object is given within the experience whether or not the experiencing subject wants to be (visually) aware of this object. When I see a table in front of me, I am not forced to believe that there is a table, I do not simply feel that the proposition “There is a table in front of me” is true, and I am not assured of the proposition's truth in the way I trust the testimony of a reliable person. However, the *presence* of the table is forced upon me in the sense that I am visually aware of the table no matter whether I want to be. I do not claim that the aforementioned authors would disagree, but their characterizations and the terminology they use are misleading.

Accordingly, my supplementation of mentalist evidentialism differs from the ones offered by Conee and Feldman and McCain in that I replace the explanationist principle CFME4 with PCEJ.

CFME1 (evidentialism): Evidence determines justification.

CFME2 (mentalism): One's justification supervenes on one's mental states.

CFME3 (experience‐first epistemology): One's experiences are one's ultimate evidence.

PCEJ: Certain experiences have a distinctive, justification‐conferring phenomenology, and if an experience E has such a justification‐conferring phenomenology with respect to proposition *p*, E, by virtue of its phenomenology, provides immediate prima facie justification for believing that *p*.

These four principles comprise my phenomenological mentalist evidentialism (PME). In the next section, I motivate PME by contrasting its virtues with the shortcomings of EME.

## 6 VIRTUES OF PME

The greatest virtue of PME is how straightforwardly it accounts for experiential justification. This is true for everyday cases (cf. section 6.4), as well as for popular epistemic thought experiments such as the new evil demon problem (cf. section 6.1). Furthermore, a phenomenological approach towards experiential justification fits well with and is supported by certain results of empirical research, particularly in experimental psychology (cf. Berghofer, forthcoming). The objective of this section is to contrast the virtues of PME with the shortcomings of EME that we discussed in section 4.

### 6.1 PME is internalist

PME states that experiences are our ultimate justifiers and that justification‐conferring experiences gain their justificatory force simply by virtue of their distinctive presentive phenomenology. PME is mentalist because it states that one's mental states, namely one's experiences, are one's ultimate justifiers. However, it is the phenomenological element that makes PME truly internalist. Its commitment to PCEJ implies that experiences gain their justificatory force by virtue of their distinctive phenomenology. This means that the epistemically relevant factor that makes experiences justifiers is located within the experiences themselves. This fits well with how Conee and Feldman lay out the basic idea of internalism: “internalism is the view that a person's beliefs are justified by the things that are internal to the person's mental life” (Conee & Feldman, 2004, 55).

Concerning experiential justification, we thus make the following distinction:

Internalist conception of experiential justification: Experiences of type T are justifiers because of factors *internal* to experiences of that type.

Externalist conception of experiential justification: Experiences of type T are justifiers because of factors *external* to experiences of that type.

A factor F is internal to experiences of type T iff (i) F is a property of experiences of T and (ii) F is part of the mental life of the person who is having the experience. Reliability, for instance, is an external factor. If you hold that experiences of a certain type are justifiers because they are reliable, you are an externalist concerning experiential justification of that type. An experience's phenomenology, on the other hand, is an internal factor. If you hold that experiences of a certain type are justifiers because of their distinctive phenomenology, you are an internalist concerning experiential justification of that type. Thus, PME – as opposed not only to reliabilism but also to EME – is internalist with respect to experiential justification.

To motivate PME, consider a variant of the new evil demon problem.

S1 and S2 are mental duplicates. Now, S1 and S2 are undergoing a perceptual experience as of a black laptop in front of them. The perceptual experience of S1 and the perceptual experience of S2 are phenomenologically indistinguishable. However, while S1's experience is veridical, S2's experience is hallucinatory.

Many epistemologists share the intuition that the perceptual experience of S1 and the perceptual experience of S2 are equal in their justificatory force concerning the proposition “there is a black laptop.” PME has a straightforward explanation: Because perceptual experiences justify by virtue of their distinctive phenomenal character, experiences with the same character justify the same propositions to the same degree. There is some general agreement that thought experiments such as the new evil demon problem speak in favour of mentalism defined as follows: “If any two possible individuals are exactly alike mentally, then they are alike justificationally, e.g., the same beliefs are justified for them to the same extent” (Conee & Feldman, 2004, 56).

Although I agree that mentalism is supported by the new evil demon problem, I believe that there is a principle that is more specific and thoroughly internalist that is even better supported by examples like the one above. I call this principle *phenomenological internalism*. Paralleling Conee and Feldman's characterisation of mentalism, I define phenomenological internalism as follows:

Phenomenological internalism: If two experiences are exactly alike phenomenologically, then they are alike justificationally, e.g., they justify the same beliefs to the same degree.

In this context, Duncan Pritchard states:

“Finally, I take it that the thesis that underlies (NEG) [i.e., the new evil demon problem] is the following:

DISC. If the experiences had by S and S* are indiscriminable then S and S* will not differ in the degree of epistemic justification that they have for their beliefs.”^13^ (Pritchard, 2011, 238)

Of course, mentalism and phenomenological internalism are perfectly consistent. PCEJ does not logically imply phenomenological internalism, but both principles fit each other perfectly. Accordingly, I complement my version of a phenomenological mentalist evidentialism as follows:

CFME1 (evidentialism): Evidence determines justification.

CFME2 (mentalism): One's justification supervenes on one's mental states.

CFME3 (experience‐first epistemology): One's experiences are one's ultimate evidence.

PCEJ: Certain experiences have a distinctive, justification‐conferring phenomenology, and if an experience E has such a justification‐conferring phenomenology with respect to proposition *p*, E, by virtue of its phenomenology, provides immediate prima facie justification for believing that *p*.

Phenomenological Internalism: If two experiences are exactly alike phenomenologically, then they are alike justificationally, e.g., they justify the same beliefs to the same degree.

It is to be noted that PME is internalist but is not in danger of leading to a vicious regress of justification. This is because PME does not demand that the subject must be aware that the experience she is undergoing justifies by virtue of its distinctive phenomenology. The experience must have a justification‐conferring phenomenology, but the subject does not need to be aware that the experience is a source of justification. Accordingly, PME does not invite the regress problem that threatens traditional forms of access internalism (cf. Bergmann, 2006, chapter 1) as well as EME (cf. Appley & Stoutenburg, 2016). Still, PME is clearly internalist as it locates an experience's justificatory force within the experience itself. It is not external factors such as truth or reliability but the internal factor of the experience's phenomenology that is epistemically relevant.

### 6.2 PME is foundationalist

Foundationalism is the view that justified beliefs are either non‐inferentially justified or ultimately justificatorily dependent upon non‐inferentially justified beliefs. According to PME, experiences are (i) a source of *immediate* justification and (ii) the *ultimate* source of justification. This means that a belief is justified iff it is either immediately experientially justified or inferentially justified such that the chain of justification can be traced back to the subject's experiences. S is *immediately experientially* justified in believing *p* iff S is having an experience that has a justification‐conferring phenomenology with respect to *p*. This experiential justification is defeasible prima facie justification. This means that PME is not committed to strong or classical foundationalism but amounts to a form of moderate foundationalism that rejects the requirement that immediate justification must be infallible (cf. BonJour, 1985, 26). S is *inferentially* justified in believing *p* iff S believes *p* on the basis of a set of propositions q_1_‐q_n_ such that (i) S is already justified in believing q_1_‐q_n_ and (ii) *p* is inferred by S from q_1_‐q_n_ in a legitimate way. It is to be noted that *inferred* does not necessarily mean “consciously inferred.” We need to distinguish between epistemic immediacy and psychological immediacy. A basic belief, a belief that is epistemically independent of one's other beliefs, is epistemically immediate. No (conscious or unconscious) inference from other beliefs is necessary for this belief to be justified. A belief is psychologically immediate if no *conscious* reasoning has taken place in order to form this belief. A belief that is psychologically immediate is not necessarily epistemically immediate.

Versions of PME, then, differ in what types of experiences they regard as exhibiting a justification‐conferring phenomenology and in what types of inferential reasoning they allow. Concerning types of justification‐conferring experiences, typical examples are perceptual experiences, introspective experiences, and intuitional experiences. Concerning types of legitimate reasoning, typical examples are deductive, inductive, and abductive reasoning.

Of course, this is not the place to discuss which types of experiences should be regarded as immediately justifying and which types of inferential reasoning should be allowed. I only wish to emphasise that PME (i) offers a straightforward foundationalist account that is committed to the plausible assumption that experiences are justifiers, (ii) explains *why* experiences are justifiers, and (iii) specifies with respect to which propositions an experience is immediately justifying.

### 6.3 No overintellectualization

PME offers a straightforward commonsense picture of experiential justification. Consider cases of perceptual justification. We all agree that we have perceptual experiences and that these perceptual experiences have a distinctive phenomenology. Also, there is some agreement that experiences justify such that when you have a perceptual experience as of a black laptop, then you are prima facie justified in believing that there is a black laptop. Certain accounts of experiential justification, such as EME, pose some kind of higher‐order requirements on justification that make it impossible or difficult for unreflective adults, children, or higher animals to have any kind of experiential justification or knowledge. Furthermore, even if we consider cases of reflective adults, such accounts often lead to counter‐intuitive results – or it is unclear how such an account would handle concrete cases. Let us say a person S has a perceptual experience as of a black laptop in front of her, but S also has the disposition to form the seeming that the best explanation for her evidence, particularly including her laptop experience, is the assumption that she is a brain in a vat and that this experience is induced to her by evil scientists. Is this scenario possible? If so, with respect to which propositions has this experience as of a black laptop justificatory force? The explanationist account seems to imply that in this scenario the person is only justified in believing that she is a brain in a vat and not even prima facie justified in believing that there is a black laptop in front of her. This is counter‐intuitive or at least it is far from a commonsense approach towards experiential justification.

According to my PME, any experience that has a presentive phenomenology with respect to *p* has justificatory force with respect to *p*, and any subject that has such an experience is prima facie justified in believing that *p*. Of course, this justification may be defeated by background beliefs or by further experiences. Then the subject is prima facie but not ultima facie justified in believing that *p*. Say a subject has a perceptual experience as of a pink elephant, but she also knows that she has just taken a drug that causes hallucinatory experiences as of pink elephants. In this case, her experience provides her with prima facie justification for believing that there is a pink elephant, but this justification is defeated by her background knowledge. I take this to be exactly the result one should expect from a plausible approach towards experiential justification.

### 6.4 Degrees of justification made easy

Assume you are walking down the street, having a visual experience as of a human being in front of you. The object you are looking at is far away, your visual experience presents the object vaguely and indistinctly. As you are approaching, the object appears to you more and more clearly and distinctly. At some point, you have a perfectly clear and distinct experience as of a male person with short brown hair, wearing a blue shirt and black glasses, looking at his smartphone. By approaching the person, you became more and more justified in believing the proposition “there is a human being.” For an explanationist or a reliabilist account, it may be difficult to pin down how exactly experiential justification increases in this example. This is because these accounts do not link an experience's justificatory force to its phenomenal character; thus, there remain further factors to be specified (e.g., the background beliefs of the person or the success rate of the person's experiences).

In our phenomenological picture, however, degrees of experiential justification can be straightforwardly explained. Different experiences can differ in the degree of justification they provide because they can differ in their respective phenomenology. Experiences do not either have or not have a justification‐conferring phenomenology; they can have it in a more or less pronounced way. The more pronounced, the more justification they provide. Thus, if two experiences differ in how clearly and distinctly they present their objects, they differ in their justificatory force simply because an experience's justificatory force is determined by how clearly and distinctly it presents its object.

To elucidate why a clear and distinct experience has more justificatory force than a vague and obscure one, we do not need to tell some story about explanatory coherence or about reliability. All we need to point out is how the respective experiences present their objects. This is why a phenomenological supplementation of mentalist evidentialism provides the most straightforward account of experiential justification.

### 6.5 Experiential justification is grounded in non‐normative terms!

In the previous subsection, we discussed how the phenomenological difference between a clear and distinct experience and a vague and obscure one is linked to the epistemological difference concerning how much justificatory force the respective experiences exhibit. The thesis of this paper is that this phenomenological–epistemological parallelism is no coincidence. As Chudnoff has recently put it, “the phenomenology grounds the epistemology” (Chudnoff, 2016, 117). Experiences justify by virtue of their presentive phenomenology, and they justify precisely those propositions with respect to which they have a presentive phenomenology. To say that the phenomenology grounds the epistemology implies that the epistemic evaluative is grounded in the descriptive non‐normative.

It is a virtue of PME to account for experiential justification in entirely descriptive, non‐epistemic terms. To assess the justificatory force of an experience, all we need to know is what it presents and how it presents it. We only need to know about the details of its phenomenal character. This leads to a truly internalist conception of experiential justification, according to which an experience's justificatory force is determined by factors that are internal to the experience. What is more, the determining factor is a descriptive, non‐normative factor. This is a further advantage PME has over EME because in the case of EME it is less clear whether or how the epistemic evaluative could be grounded in something non‐normative.

There is one further advantage of PME that I would like to address. In this paper, we have been concerned with propositional justification. Our principle of propositional experiential justification is PCEJ.

PCEJ: Certain experiences have a distinctive, justification‐conferring phenomenology, and if an experience E has such a justification‐conferring phenomenology with respect to proposition *p*, E, by virtue of its phenomenology, provides immediate prima facie justification for believing that *p*.

What is expected from a comprehensive epistemic theory is to provide a clear transition from propositional to doxastic justification. For PME, this is no problem. PCEJ handles propositional justification, and all that is required from the subject to gain doxastic justification is that *the respective belief is based on the respective justification‐conferring experience*. When your experience presents you with a black laptop, then you have propositional justification for believing that there is a black laptop. When you believe that there is a black laptop and your belief is based on your experience presenting the black laptop to you, then your belief is doxastically justified. For an explanationist account, things are not that simple. This is obvious when you consider cases in which it is *not* the case that you are aware of the fact that *p* is part of the best explanation for why you are having the evidence you have (but you have the disposition to have the seeming that this is so). Because your belief can hardly be based on the *disposition* to have a seeming, there is no straightforward transition from propositional justification to doxastic justification.^14^

## Footnotes

^1^
At least this is how internalist and externalist positions are usually contrasted (cf., e.g., Steup, 2016, section 2.3). In section 4.1, we will see that certain positions that seem to be externalist are consistent with (all the above‐mentioned versions of) mentalism.

^2^
In fact, below we shall see that Conee and Feldman call their position “a non‐traditional version of coherentism.” However, most epistemologists who, similarly to Conee and Feldman, stress the epistemic significance of experiences subscribe to a form of moderate foundationalism. The foundationalist alternative that I introduce in section 5 should be more attractive to philosophers with foundationalist inclinations than the account offered by Conee and Feldman. This will be discussed in detail in sections 4.2 and 6.2.

^3^
The explanationism formulated in Conee & Feldman, 2008 remains sketchy. The present formulation of explanationism is based on more elaborated versions as they are discussed in Appley & Stoutenburg, 2016; Byerly & Martin, 2014; McCain, 2014, 2018a.

^4^
Because phenomenological approaches to experiential justification are comparatively new contenders in current debates, there are not many works explicitly objecting to them. However, one objection can be found in Teng, 2018. I have defended the phenomenological account against this line of reasoning elsewhere, cf. Berghofer, 2020a.

^5^
This view that one's evidence supervenes on one's non‐factive mental states is often referred to or associated with *evidential internalism*. Evidential internalism has been forcefully defended by Silins, who introduced it as the view that internal twins have the same evidence, even if one of them is radically deceived (Silins, 2005). Williamson (2000) and Kelly (2008) discuss this view under the label “phenomenal conception of evidence.” It plays an important role in current debates and constitutes a chief target of externalists (cf. Fratantonio & McGlynn, 2018). I subscribe to *evidential internalism*. Importantly, however, I believe that evidential internalism suggests a *phenomenological* conception of evidence according to which the phenomenology of one's experiences plays a central epistemological role. What this means is explained in section 5. At this point I only want to emphasise that Williamson, perhaps the chief opponent of *evidential internalism*, says: “That one has the same evidence in the good and bad cases is a severe constraint on the nature of evidence [… such that it] drives evidence towards the purely phenomenal” (Williamson, 2000, 173). Here I agree with Williamson. It is the objective of this paper to show that internalists should embrace a phenomenological conception of evidence in the precise sense introduced later in section 5.

^6^
For versions of such a conception of experiential justification, cf., e.g., Chudnoff, 2013; Huemer, 2001; Pryor, 2004.

^7^
For the objection that (Haack's version of) foundherentism collapses into (weak) foundationalism, cf. BonJour, 1997 and Tramel, 2008. For objections against weak foundationalism, cf. BonJour, 1985, 29. In contrast to traditional forms of coherentism, EME (and foundherentism in general) rejects the idea that coherence is sufficient for justification. This is because not any kind of coherence can lead to justification. EME ascribes a special role to experiences. Experiences are the ultimate evidence. Basically, it is the coherence between our experiences and the best available explanation for why we have these experiences that justifies. In short, EME denies that coherence is sufficient but states that it is necessary for justification and ascribes a special status to experiences. Coherentists (in the traditional sense) would insist that coherence is sufficient for justification and reject the special status of experiences. Foundationalists, on the other hand, would deny that coherence is necessary for justification and, typically, argue that experience is an autonomous source of epistemic justification such that beliefs based on experiences are (prima facie but properly) justified. Because EME has it that neither coherence nor experience is sufficient for justification, it remains somehow mysterious how the combination of both can lead to proper justification. Also, EME does not really offer an explanation for why experiences are special. What gives them their epistemological significance? Finally, we note that coherentists, typically, argue that it is coherence between *beliefs* that justifies. Conee and Feldman, by contrast, talk about coherence between *propositions*. It seems that all that matters to them is whether it is objectively true that the propositions in question are the best available explanation for one's experiences, regardless of whether the subject actually believes this to be the case. This might lead to further problems discussed below.

^8^
For a defence of explanationist mentalist evidentialism against this objection, cf. Lutz, 2018.

^9^
For such objections to internalism, cf., e.g., Bergmann, 2006 and Greco, 2014.

^10^
By an experience's phenomenology or phenomenal character, I understand what it is like from the first‐person perspective to undergo the experience. As Siegel puts it, “It is definitional of experience, as the term is used here, that they have some phenomenal character, or more briefly, some phenomenology. The phenomenology of an experience is what it is like for the subject to have it” (Siegel, 2016).

^11^
Cf., e.g., Husserl, 1982, 36, 44; 2008, 342 f.

^12^
As a side note, this is an important advantage over Michael Huemer's popular principle of phenomenal conservatism PC (cf. Huemer, 2001). For powerful objections to PC, cf. Markie, 2005. For discussions of Markie's objections, cf. Brogaard, 2016 and Berghofer forthcoming.

^13^
It should be mentioned that Pritchard does not subscribe to DISC or phenomenological internalism.

^14^
McCain discusses doxastic justification in terms of well‐foundedness. His characterisation of well‐foundedness is about one page long (McCain, 2014, 118). Here he states that one's evidence also consists of mental states the subject “is disposed to bring to mind when reflecting on the question of *p*'s truth” and he requires that for a belief to be well‐founded each piece of evidence must be “a direct cause” as well as “an actual cause” for the subject's believing that *p*. I do not see how mental states the subject is disposed to bring to mind can fulfil the role of being a direct and an actual cause of one's belief.
